# Modulating Cerebrospinal Fluid Composition in Neurodegenerative Processes: Modern Drug Delivery and Clearance Strategies

**DOI:** 10.3390/ijms262311541

**Published:** 2025-11-28

**Authors:** Elizaveta A. Dutysheva, Anastasiya V. Zaerko, Mikita A. Valko, Ekaterina O. Antipina, Sergey M. Zimatkin, Boris A. Margulis, Irina V. Guzhova, Vladimir F. Lazarev

**Affiliations:** 1 Institute of Cytology, Russian Academy of Sciences, St. Petersburg 194064, Russia; 2 Department of Histology, Cytology and Embryology, Grodno State Medical University, 230009 Grodno, Belarus; 3 Scientific Laboratory, Grodno State Medical University, 230009 Grodno, Belarus; 4 Department of Neurology and Neurosurgery, Grodno State Medical University, 230009 Grodno, Belarus

**Keywords:** cerebrospinal fluid, neurodegeneration, biomarkers, drug delivery, blood–brain barrier, bispecific antibodies, nanoparticle-based therapy, biomarkers, protein aggregates

## Abstract

Neurodegenerative diseases, traumatic brain injuries, and strokes are accompanied by the development of secondary damage—a long-term pathological cascade in which cerebrospinal fluid (CSF) plays a key role. Unlike primary damage, which is acute, secondary processes can progress over months and even years, creating a therapeutic window for neuroprotection. CSF acts not simply as a passive medium but as an active mediator of the spread of cytotoxic factors—reactive oxygen species, glutamate, proinflammatory cytokines, pathological protein aggregates (Aβ, α-synuclein, tau, etc.), and exosomes—which transport toxic molecules between brain regions. These processes are exacerbated by dysfunction of the blood-brain and blood–cerebrospinal fluid barriers, leading to the accumulation of damaging agents in the CSF and accelerated neurodegeneration. This review examines the molecular mechanisms of secondary injury, the role of barrier systems in maintaining CSF homeostasis, and current therapeutic strategies aimed at modulating CSF composition. Particular attention is paid to innovative approaches to drug delivery to the central nervous system—from bispecific antibodies and nanoparticles to invasive techniques such as immunoselective CSF aspiration and nanoporous implants. The potential of CSF as a source of diagnostic biomarkers and as a therapeutic target for personalized treatment of neurodegenerative conditions is highlighted.

## 1. Introduction

The vast majority of neurodegenerative processes are accompanied by the accumulation of cytotoxic factors of various natures in the intercellular space of the brain and in the cerebrospinal fluid (CSF).

These can include protein factors such as misfolded, denatured, or oligomerized proteins and peptides, pro-inflammatory cytokines, non-protein low-molecular factors such as glutamate, calcium, and reactive oxygen species, and can also include more complex structures such as exosomes [1,2,3,4].

As neurodegenerative processes progress and a critical mass of these factors forms, they begin to significantly influence the pathogenesis of diseases, accelerating the degradation of nervous tissue. This phenomenon is well-known and is called secondary damage [5]. Secondary damage can develop over a very long period, often throughout life [6,7]. It includes a newly introduced concept of proteinjury—a pathogenic process of cerebral tissue damage mediated by proteotoxic agents circulating in CSF, which are themselves a sequel to a primary traumatic insult or neurodegenerative etiology [8].

Another significant pathological process associated with CSF is disruption of the blood–brain barrier (BBB), the blood–cerebrospinal fluid barrier, and the choroid plexus [9]. Such disturbances can also accelerate the accumulation of cytotoxic factors, inflammatory mediators, and other damaging agents in the CSF, which in turn will negatively impact the development of neurodegenerative processes. Understanding the role and function of these barriers and the choroid plexus in the accumulation of cytotoxic factors in the CSF is important for developing treatment and prevention strategies for neurological diseases associated with these processes [10].

Naturally, in recent years, numerous scientific results have accumulated describing therapeutic approaches for CSF purification and the neutralization of toxic factors [11,12,13]. Although not yet used clinically, such approaches have repeatedly demonstrated their effectiveness and potential in basic scientific research (including in vivo) [14,15].

Thus, it is safe to say that blocking cytotoxic factors in the CSF plays an important role in protecting nerve tissue, reducing inflammation, improving symptoms, and slowing the progression of neurodegenerative diseases. In this review, we examine modern possibilities and approaches in science and medicine in the context of blocking cytotoxic factors in CSF in order to increase the effectiveness of blocking neurodegenerative processes of various origins.

## 2. CSF as a Mediator of Secondary Injury

Secondary brain injury is a complex cascade of pathophysiological processes triggered after a primary stroke, traumatic brain injury (TBI), or during the progression of neurodegenerative diseases (NDDs) such as Alzheimer’s, Parkinson’s, or Huntington’s. Unlike primary injury—mechanical or ischemic, which occurs within seconds or minutes—secondary mechanisms develop gradually and can persist for weeks, months, and even years [16,17,18]. It is this duration that creates a therapeutic window for neuroprotective interventions.

CSF plays a key role in the spread and maintenance of secondary injury. As a dynamic environment bathing the brain and spinal cord, CSF not only provides mechanical protection and homeostasis but also transports biologically active molecules: from neurotransmitters and cytokines to pathological protein aggregates and extracellular vesicles. Thus, CSF acts as a systemic conductor of pathological signals, facilitating the diffuse spread of damage even to regions of the brain distant from the primary focus.

### 2.1. Oxidative Stress

**Figure 1:**
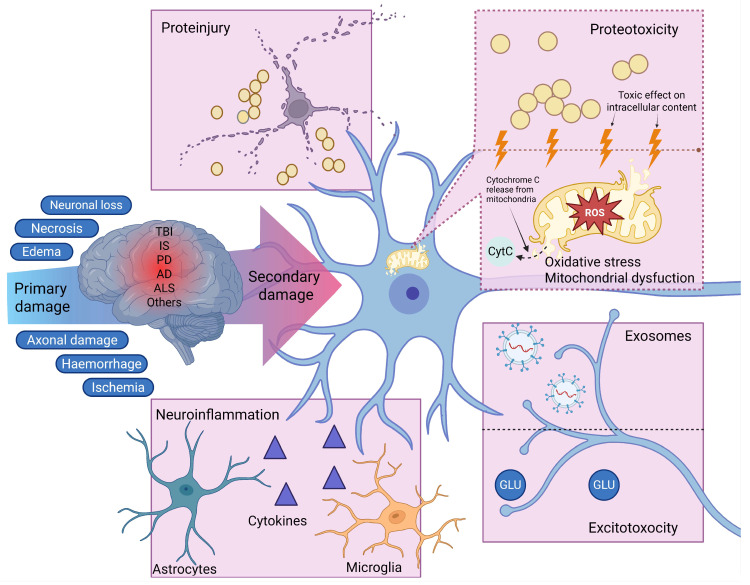
Molecular mechanisms underlying secondary brain injury mediated by CSF. The schematic illustrates how cytotoxic factors—ROS, excitotoxic glutamate, proinflammatory cytokines, pathological protein aggregates and exosomes—accumulate in the CSF following primary insults such as neurodegeneration, stroke, or traumatic brain injury. These agents propagate damage across brain regions by inducing oxidative stress, calcium dyshomeostasis, neuroinflammation, proteotoxicity, proteinjury, and intercellular transmission of pathology via exosomes, thereby sustaining a chronic cascade of secondary injury. The dashed line indicates the separation of distinct cytotoxicity mechanisms within a shared microenvironment. Created with BioRender.com. Created in BioRender. Kachkin, D. (2025) https://BioRender.com/5j4439a.

One of the early and persistent components of secondary injury is oxidative stress caused by excess reactive oxygen species (ROS) and nitrogen oxides (Figure 1). Following TBI or ischemia, mitochondrial metabolism is disrupted, with activation of NADPH oxidases, Ca^2+^-dependent NO synthases, and phospholipases, leading to the massive generation of superoxide anion, hydrogen peroxide, hydroxyl radicals, and peroxynitrite [19,20]. These compounds cause lipid peroxidation, protein carbonylation, and damage to nuclear and mitochondrial DNA [21,22].

Neurons are particularly vulnerable due to the high content of polyunsaturated fatty acids in their membranes and high oxygen consumption. Mitochondrial damage triggers the apoptotic cascade: cytochrome c release, apoptosome formation, and caspase-3 activation [23]. Simultaneously, prosurvival pathways such as PI3K/Akt are suppressed, depriving cells of the ability to compensate for stress. Oxidative damage markers, such as 8-oxoguanine, 4-hydroxynonenal, and nitrotyrosine, are detected not only in brain tissue but also in the CSF of patients with neurodegenerative diseases and post-TBI, confirming the role of CSF as a reservoir and transporter of oxidative stress [24].

### 2.2. Excitotoxicity and Disruption of Calcium Homeostasis

Excitotoxicity caused by excessive glutamate release is another early and potent trigger of secondary damage (Figure 1). During TBI, stroke, or neurodegeneration, glutamate accumulates in the extracellular space and CSF, where its concentration can exceed physiological levels by tens of times. This leads to hyperactivation of NMDA and AMPA receptors, massive influx of Na^+^ and Ca^2+^ into neurons, and subsequent disruption of osmotic and energy balance [25,26].

Excess intracellular Ca^2+^ activates calcium-dependent enzymes, such as calpains, phospholipases, endonucleases, and nitric oxide synthases, which exacerbates damage to the cytoskeleton, membranes, and DNA. Furthermore, Ca^2+^ induces the opening of mitochondrial proton pump pores (mPTPs), which exacerbates oxidative stress and promotes apoptosis. Interestingly, changes in the ionic composition of CSF, including increases in [Ca^2+^], may have systemic effects: animal experiments have shown that injection of Ca^2+^-enriched CSF reduces blood pressure, indicating a neuroendocrine function of CSF [27,28,29].

### 2.3. Neuroinflammation

Neuroinflammation is a central component of secondary injury, especially in long-term pathologies (Figure 1). Damage to the BBB during trauma or neurodegenerative diseases allows peripheral immune cells and proinflammatory mediators to penetrate the central nervous system [30,31]. Microglia are activated within the first hours after injury and begin releasing cytokines (TNF-α, IL-1β, IL-6, INF-γ) [32,33,34], chemokines, and ROS [35].

These molecules are not limited to the lesion site: they are found in the CSF and can spread throughout the ventricular and subarachnoid systems, causing “distant” inflammation. Clinical data show that IL-6 and IL-8 levels in the CSF in older adults correlate with white matter lesion volume and cognitive decline, particularly in the presence of β-amyloid (Aβ) [36]. Thus, the CSF becomes a medium for the development of a pathological feedback loop: injury—inflammation—cytokine release into the CSF—further injury [37].

### 2.4. Proteotoxic Stress and Proteinjury

Disruption of protein homeostasis is a fundamental mechanism of secondary injury in a wide variety of CNS pathologies. Under conditions of oxidative stress, excitotoxicity, and inflammation, proteins either lose their native conformation and form toxic oligomers and aggregates or experience more prominent aggregation of initially aberrant protein. Classic examples include Aβ and hyperphosphorylated tau in Alzheimer’s disease (AD), α-synuclein in Parkinson’s disease (PD), and mutant huntingtin in Huntington’s disease. However, similar aggregates are also detected after TBI or stroke, even in the absence of a genetic predisposition [12,13,38]. It’s important to distinguish between proteotoxic stress (the intracellular accumulation of misfolded proteins) and proteinjury—the extracellular toxic effect of pathological protein complexes circulating in the intercellular space and CSF [8]. Aggregates isolated from the CSF of Alzheimer’s patients or from the CSF of rats after TBI maintain cytotoxicity in neuronal and glial cultures for months. This indicates the potential for long-term effects of proteinjury, making it one of the most persistent factors of secondary injury (Figure 1).

### 2.5. The Role of CSF Exosomes in the Spread of Secondary Injury

Exosomes—extracellular vesicles 30–150 nm in diameter, secreted by virtually all CNS cells and present in significant quantities in the CSF—are of particular importance in the pathogenesis of secondary injury. Exosomes play a dual role: under physiological conditions, they participate in intercellular communication, neurotrophic signaling, and the clearance of excess proteins; however, in pathological conditions, they become vectors for the spread of toxic agents participating in secondary damage processes [39] (Figure 1).

In neurodegenerative diseases and post-TBI, CSF exosomes are enriched with pathological proteins: phosphorylated tau, oligomeric α-synuclein, Aβ, as well as proapoptotic miRNAs and inflammatory cytokines [32,33,40,41]. These vesicles protect their contents from degradation, ensuring the stable delivery of toxic molecules to distant neuronal populations. Experiments have shown that exosomes isolated from the CSF of AD patients induce tau aggregation in healthy neurons in vitro, showing an effect reminiscent of the “prion-like” spread of pathology [42,43].

Furthermore, exosomes from microglia and astrocytes during inflammation carry adhesion molecules on their surface and activate TLR receptors on neighboring cells, enhancing neuroinflammation. Recent studies also indicate that exosomes can modulate BBB permeability and promote the infiltration of peripheral immune cells [44,45]. Thus, CSF exosomes act not simply as passive carriers, but as active regulators of the pathological cascade, facilitating the interregional transmission of secondary damage signals.

Conclusion. CSF is not an inert fluid, but a dynamic biological system that integrates and disseminates key mechanisms of secondary injury: oxidative stress, excitotoxicity, neuroinflammation, proteotoxicity, and intercellular transmission of pathology via exosomes. CSF plays a particularly significant role in promoting long-term extracellular protein toxicity (proteinjury) and exosome-mediated “infection” of healthy neurons. These data highlight the therapeutic potential of modulating CSF composition—specifically, by enhancing the clearance systems for pathological proteins and exosomes, which will be discussed in the next section.

## 3. Barrier Systems Regulating Homeostasis in the CSF

During normal nerve cell activity, numerous protein molecules and cellular metabolic products appear in the interstitial space. On the other hand, protein complexes circulating in the blood and lymph should not penetrate brain tissue. During neurodegenerative processes, oligomers and protein aggregates appear in the interstitial space. These cellular waste products are utilized and removed by protein clearance systems, which ensure their transport into the CSF and then into the blood and lymph. In pathological conditions, the clearance system fails, resulting in the accumulation of aggregates both in brain tissue and in the CSF. The accumulation of such structures in the interstitial space causes proteinjury (cell death mediated by extracellular toxic protein species).

**Figure 2:**
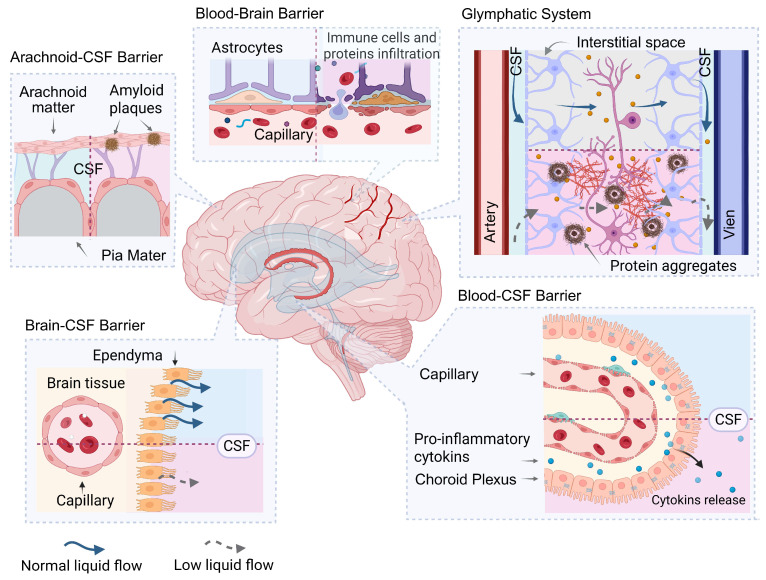
Barrier systems regulating CSF homeostasis. The figure depicts the major interfaces that control molecular exchange between blood, brain parenchyma, and CSF: the blood–brain barrier, formed by endothelial cells with tight junctions; the blood–cerebrospinal fluid barrier, located at the choroid plexus epithelium; the ependymal layer lining the ventricles, which separates CSF from the interstitial fluid of the brain; Glymphatic system, that provided protein clearance and the pia and arachnoid mater, which form the leptomeningeal barrier at the brain surface. These structures collectively maintain CSF composition, facilitate clearance of metabolic waste, and protect the CNS—but their dysfunction in neurodegenerative and acute brain injuries contributes to the accumulation of cytotoxic factors in the CSF, exacerbating secondary damage. The dashed line separates each diagram into two domains: normal physiological conditions (blue/gray background) and pathological conditions (pink background). Created with BioRender.com. Created in BioRender. Kachkin, D. (2025) https://BioRender.com/n1vh9dw.

At the same time, in addition to the removal of biomolecules from brain tissue into the CSF and beyond, there is a counter-current flow from the blood into the CSF and then into brain tissue. This process is also regulated by the same barrier systems of the body [46] (Figure 2).

Thus, protein clearance systems are important in the context of the progression of neurodegenerative pathologies for several reasons:

- isolating the CSF from cytotoxic proteins, proinflammatory cytokines, and other biomolecules that can damage brain tissue when released into the CSF;
- clearing the CSF of cytotoxic proteins and their complexes, and other biological factors that cause secondary brain damage;
- acting as a target for cytotoxic factors contained in the CSF or interstitial space, including pathogenic protein complexes, i.e., as a target for factors that cause secondary damage, including proteinjury.

Below, we will examine in more detail the existing components of the protein clearance system and the known features of their functioning in various neurodegenerative conditions.

### 3.1. Blood–Brain Barrier

The BBB—is a key structural and functional element that provides a specific protein environment for nervous tissue. Its primary role is to protect the brain from toxins, pathogens, and protein factors circulating in the blood, as well as to remove metabolites from nervous tissue into the systemic circulation (Figure 2).

Disruption of the BBB integrity is a characteristic feature of both acute CNS injuries (trauma, stroke) and chronic neurodegenerative diseases [47]. Experimental models of TBI in mice demonstrate disruption of the BBB and partial loss of immune privilege in the brain [48]. In patients, signs of BBB damage (e.g., fibrinogen and IgG leakage into brain tissue) can persist for more than a year after injury [49] and are associated with an increased risk of dementia [50]. Moreover, increased BBB permeability to proteins is considered an early biomarker of cognitive impairment [51,52]. In mice, chronic barrier disruption promotes Aβaccumulation in neurons, highlighting its role in the development of secondary, including protein-mediated, damage [53]. The inverse relationship has also been confirmed: neurodegenerative processes themselves impair BBB function. Thus, α-synuclein aggregates reduce the viability of BBB endothelial cells in vitro [54], and loss of TDP43 in the endothelium of mice with an ALS/frontotemporal dementia model increases barrier permeability [55]. Similarly, Aβ increases BBB permeability, in particular through the induction of RAGE expression in endothelial cells [56,57].

Thus, BBB damage in neurodegenerative conditions inevitably alters the composition of the CSF, primarily its protein profile [58]. These changes can both initiate and aggravate pathological processes: disruption of the BBB facilitates the penetration of cytotoxic protein complexes (proteinjury), which, in turn, further damage the barrier, forming a vicious circle.

### 3.2. Blood-CSF Barrier

The blood–cerebrospinal fluid barrier or Blood-CSF barrier is a key component of the central nervous system’s defense system, complementing the BBB (Figure 2). Blood-CSF barrier, also referred to in the literature as the BCSFB, is based on the choroid plexus, a specialized structure located in the cerebral ventricles and consisting of fenestrated capillaries covered by a layer of polarized epithelial cells connected by tight junctions. This structure regulates exchange between blood and CSF, ensuring its production and maintaining its unique protein and ion composition [59,60,61].

Unlike the BBB, which is dominated by selective transport of nutrients to neurons, Blood-CSF barrier is actively involved in the global delivery of hormones, growth factors, peptides, and other signaling molecules into the CSF, as well as the removal of metabolites from the CNS [62,63]. Normally, transport proteins, immunoglobulins, cytokines, and neurotrophic factors primarily enter the CSF through the choroid plexus, emphasizing its role not only in barrier function but also in immune and metabolic regulation of the brain environment [64].

In neurodegenerative diseases, particularly AD, Blood-CSF barrier function is impaired. Increased barrier permeability, decreased CSF production and turnover, and accumulation of Aβ and proinflammatory cytokines in the choroid plexus have been observed [65,66,67]. Aβ oligomers can induce the expression of matrix metalloproteinases in epithelial cells, which further damage the barrier [68]. These changes correlate with the severity of cognitive impairment and contribute to chronic neuroinflammation [69,70]. Similar disturbances occur during acute CNS injury, e.g., TBI, stroke, or hyperthermia leading to cerebral edema and immune cell infiltration into the CSF [71,72]. Experimental models have shown that neurotrophic factors (BDNF, GDNF, cerebrolysin) can restore integrity, reduce edema, and improve neurological outcome [73,74,75].

Thus, the choroid plexus and Blood-CSF barrier represent a dynamic interface system critical for CSF protein homeostasis. Their dysfunction as a cause and consequence is implicated in the pathogenesis of a wide range of neurological diseases, including neurodegeneration and acute brain injury.

### 3.3. CSF-Brain Barrier

Although the choroid plexus is considered to be the main source of CSF, evidence has recently begun to emerge that ependymal cells lining the cerebral ventricles, previously considered functionally inert, also perform barrier functions and participate in both the secretion and reabsorption of CSF, forming a CSF-Brain barrier [76]. Ependyma is of glial origin and is characterized by the presence of tight junctions that ensure selective penetration of molecules from the parenchyma and adjacent vessels into the CSF. The tight junctions between ependymal cells are formed by cadherins [77], claudins [78], connexins [79] and others, and changes in the permeability of the transependymal barrier may play an important role in pathological conditions of the brain (Figure 2).

Hydrocephalus often accompanies neurodegenerative conditions such as AD [80], PD [81], chronic traumatic encephalopathy [82] and others. Postmortem examination of human fetuses with severe hydrocephalus revealed a mutation in the MPDZ gene, which encodes the ependymal tight junction component [83]. In the third and fourth ventricles, irregular rosette-like structures formed by ependymal cells were found, disrupting the integrity of the barrier.

Among other things, the ependyma plays a crucial role in CSF circulation. A mouse model of TBI revealed a reduction in the number of ependymal cilia in the animals’ ventricles. This change lasted for up to three weeks and was accompanied by a slowdown in CSF circulation [84].

Despite the lack of data confirming the functions of CSF-Brain barrier, the importance of this brain barrier is emerging in both health and disease. For example, one study demonstrated that ependymal cells are involved in the removal of Aβ from the CSF and its lysosomal degradation via the RAGE receptor in patients who have suffered cardiac arrest [85].

### 3.4. Arachnoid and Pia Mater

The arachnoid and pia mater form the subarachnoid space and leptomeningeal barrier, which is filled with CSF, and play a crucial role in maintaining its protein homeostasis. The pia mater adheres tightly to the brain’s surface and serves as a barrier between nervous tissue and CSF, regulating the exchange of proteins and other molecules between them (Figure 2). The arachnoid mater, which separates the CSF from the dura mater, functions as a passive filter, participating in the selective transport of substances between surrounding tissues and the CSF [86,87].

Thus, both membranes work together to maintain a stable protein composition of the CSF, which is critical for normal CNS function. However, in neurodegenerative diseases, particularly AD, Aβ deposits are found in the arachnoid membrane, which can disrupt its structure and barrier properties [88,89].

Despite the obvious importance of these membranes in the pathogenesis of neurodegeneration, data on the mechanisms of protein transport across the arachnoid membrane remain extremely limited. Traditionally, this interface is considered part of the BBB, although its specific role in regulating CSF composition requires further study.

### 3.5. Glymphatic System

Although not a barrier in the classical sense, the glymphatic system plays a pivotal role in maintaining CSF and interstitial fluid (ISF) homeostasis by actively clearing metabolic waste from the brain parenchyma. First described in 2012 by Nedergaard and colleagues, this paravascular network facilitates the directional flow of CSF from periarterial spaces into the brain interstitium, where it mixes with ISF and solutes—including pathological protein aggregates such as Aβ and tau—before draining into perivenous channels for eventual removal from the CNS [90,91].

The efficiency of this convective clearance heavily depends on astrocytic aquaporin-4 water channels, which are polarized at perivascular endfeet and enable rapid water exchange between CSF and ISF [92,93]. Critically, glymphatic activity declines with age and is further compromised in neurodegenerative conditions and after acute brain injury [94,95]. This dysfunction contributes to the accumulation of neurotoxic proteins not only in the interstitial space but also in the CSF, thereby exacerbating secondary injury mechanisms such as toxic extracellular protein damage (proteinjury) and neuroinflammation. Thus, while the glymphatic system does not act as a selective barrier like the BBB or Blood-CSF barrier, it serves as a dynamic clearance infrastructure that directly influences CSF composition and, consequently, the progression of neurodegenerative pathology.

## 4. CSF as an Object for Diagnostics

CSF represents a critical component of the body’s internal environment for the diagnosis of nervous system pathologies in general, and neuropathologies in particular. The investigation of CSF as a source of biomarkers for major neurodegenerative diseases is particularly promising. This is relevant for conditions such as AD, PD and atypical parkinsonian syndromes, ALS, Huntington’s disease, and Creutzfeldt-Jakob disease. Currently, research distinguishes between primary and secondary biomarkers, as well as more and less promising candidate markers. Changes in the concentration or quantity of both primary and secondary biomarkers in CSF are directly associated with the pathogenesis of each disease.

In AD, a decrease in CSF Aβ peptide levels compared with healthy individuals is highly indicative of pathology [96]. This reduction reflects the deposition of Aβ42, a key metabolite in the pathogenesis of the disease. Elevated levels of total and phosphorylated tau are also diagnostically valuable, corresponding to another pathogenic mechanism: tau hyperphosphorylation, formation of neurofibrillary tangles, and disintegration of the neuronal transport system [97].

Current literature reports over 90 detectable compounds in CSF that may serve as additional biomarkers for AD. However, the strength of evidence for most of these markers remains limited due to small sample sizes, inconsistent clinical findings, and low specificity [98,99]. Nonetheless, several CSF changes demonstrate high specificity for AD and are considered candidates for primary biomarkers. These include increased levels of light and heavy chains of neurofilament proteins (NfL and NfH) and glial fibrillary acidic protein (GFAP), reflecting neuronal ultrastructural damage [100].

Other candidate biomarkers include BACE1 (β-site amyloid precursor protein-cleaving enzyme 1), a direct contributor to Aβ formation; APP (amyloid precursor protein), the Aβ precursor; VSNL1 (visinin-like protein 1), a calcium-binding protein released during neuronal injury; various inflammatory mediators; and decreased levels of neurotrophic factors [101].

With regard to PD, the division of CSF biomarkers into primary and secondary is not entirely accurate, as there are currently no widely accepted and highly specific CSF markers for this condition [102]. Therefore, it is more appropriate to discuss the “most and least promising” CSF biomarkers for PD. The challenges are similar to those described for AD, with an additional complication arising from the similarity of many CSF marker changes in PD and other neurological disorders classified as atypical parkinsonian syndromes, including diffuse Lewy body disease, multiple system atrophy, progressive supranuclear gaze palsy, and corticobasal syndrome [103].

One of the most promising CSF biomarkers for PD is α-synuclein, which is typically reduced in patients compared with healthy individuals, but shows similar levels in diffuse Lewy body disease and multiple system atrophy [104]. Combined measurement of CSF α-synuclein, phosphorylated and total tau, Aβ42, and neurofilament light chains may provide a robust method for differential diagnosis among PD, atypical parkinsonian syndromes, AD, and other neurodegenerative conditions [105].

Longitudinal studies indicate that CSF α-synuclein levels increase as PD progresses [106]. Measurement of total α-synuclein, its oligomeric fraction and phosphorylated forms is also promising; one study reported a correlation between worsening motor function and changes in the ratio of oligomeric to total α-synuclein [107]. The application of RT-QuIC (real-time quaking-induced conversion) technology, originally developed for prion proteins in Creutzfeldt–Jakob disease, has demonstrated high sensitivity for detecting α-synuclein in CSF from PD patients [108].

Another key component of Lewy bodies, neurofilament light chains (NfL), is found in similar amounts in the CSF of Parkinsonian patients and healthy individuals. However, NfL levels in combination with other CSF markers are valuable for distinguishing PD from atypical parkinsonian syndromes, as elevated NfL is more characteristic of the latter [109]. While CSF Aβ42 is consistently reduced in PD patients with cognitive impairment or dementia, levels of total and phosphorylated tau generally remain within normal ranges and do not reliably differentiate PD dementia from non-demented Parkinson’s disease [110].

The protein deglycase DJ-1 (Parkinson’s disease protein 7) is another potential CSF biomarker for PD, although studies report conflicting results, with some showing increased and others decreased CSF levels [103].

Overall, these CSF marker changes correspond to PD pathogenesis: α-synuclein and Aβ42 aggregation and deposition in neurons reduce their CSF levels, while tau increases due to hyperphosphorylation.

For atypical parkinsonian syndromes and other neurodegenerative conditions, analysis of biomarker panels rather than single markers is considered more informative. For example, in dementia with Lewy bodies, which clinically overlaps PD and AD, simultaneous assessment of Aβ40 and Aβ42, tau, α-synuclein, and the tau/α-synuclein ratio is recommended. Despite the existence of methods capable of discriminating between diseases on the basis of a single marker, as exemplified by the seed amplification assay (SAA) for Parkinson’s disease and multiple system atrophy [111], individual marker assessment can yield misleading results due to the overlap of biomarker changes among different pathologies [112,113]. Similar conclusions apply to multiple system atrophy, progressive supranuclear gaze palsy, and corticobasal syndrome. Neurofilament proteins NfL and NfH are consistently identified as among the most promising biomarkers for atypical parkinsonian syndromes [113,114]. Additionally, recent studies report the successful use of mRNA panels as a diagnostic approach for atypical parkinsonian syndromes using CSF analysis.

For ALS, as with PD, there are currently no widely accepted and reliable CSF biomarkers. The most promising candidates are the light and phosphorylated heavy chains of neurofilament proteins, whose levels in the CSF of amyotrophic lateral sclerosis (ALS) patients are significantly higher than in healthy individuals or patients with other neurodegenerative diseases, and which correlate directly with disease progression. Evidence also supports the successful diagnosis of ALS through combined measurement of phosphorylated neurofilament heavy chains and complement 3 proteins in the CSF [115].

Mutated SOD1 and TDP-43 proteins, which are implicated in ALS pathogenesis, are also considered potential CSF biomarkers [116]. While studies of SOD1 levels in CSF have produced conflicting results, CSF levels of NfL—but not TDP-43—have consistently demonstrated utility in differentiating ALS from mimics such as Guillain–Barre syndrome [117,118].

Other molecules, including metalloproteinases (particularly MMP-2 and MMP-9) and tau, show distinctive dynamics in ALS patients’ CSF and are considered promising biomarkers due to their roles in BBB disruption and neuronal ultrastructural damage, respectively [103]. Combinations of markers are preferred for CSF-based ALS diagnostics; for example, a panel containing transthyretin, cystatin C, and the carboxy-terminal fragment of neuroendocrine protein 7B2 has demonstrated high diagnostic accuracy [119].

In Huntington’s disease, the mutant huntingtin protein, detectable in the CSF of affected patients but absent in healthy individuals, is considered a biomarker. Its CSF levels positively correlate with tau and NfL levels, as well as with disease progression [120]. Additionally, elevated CSF ubiquitin levels are proposed as a complementary biomarker for Huntington’s disease [121].

For Creutzfeldt–Jakob disease, the 14-3-3 protein is a key CSF biomarker, enabling prediction of disease presence and, when assessed alongside MRI, electroencephalography, and clinical history, differential diagnosis of other neurodegenerative disorders [122]. Tau is also considered a potential CSF biomarker for Creutzfeldt–Jakob disease, with elevated levels observed in the majority of patients with both sporadic and hereditary forms, correlating with disease progression. Assessment of total and phosphorylated tau, in combination with 14-3-3 protein levels, is recommended for accurate differential diagnosis between Creutzfeldt–Jakob disease and AD [103,123].

RT-QuIC, a technology originally developed for the diagnosis of Creutzfeldt-Jakob disease, has proven to be highly effective for this purpose. In this context, it is employed to detect in the CSF PrPSc—the prion protein directly responsible for the pathogenesis of the disorder [124].

The information presented above on alterations in the levels of substances proposed as CSF biomarkers for various neurodegenerative diseases can be summarized in tabular form (Table 1). These data once again underscore the importance of analyzing changes in multiple CSF components to ensure high-quality laboratory assessment and accurate diagnosis. Such an approach enables not only early detection of neuropathological processes but also reliable differential diagnosis. Furthermore, it highlights the necessity for continued research aimed at identifying new biomarkers and validating existing candidate compounds.

## 5. Modern Methods of Therapeutic Agents Delivering into the CSF

Understanding the key role of CSF as a mediator of secondary injury and a reservoir of cytotoxic agents from reactive oxygen species and glutamate to pathological protein aggregates and proinflammatory cytokines opens up a direct therapeutic opportunity: modulating CSF composition could become an effective means of neuroprotection in neurodegenerative diseases, TBI, and stroke. However, the blood–brain and blood–cerebrospinal fluid barriers, which protect the cerebrospinal space from the systemic circulation, simultaneously prevent the penetration of the vast majority of therapeutic agents from the periphery. Moreover, even when these barriers are successfully overcome, drug distribution within the brain and CSF is limited by diffusion gradients and rapid clearance.

The molecular profile of CSF—including levels of Aβ, tau, α-synuclein, neurofilaments, and inflammatory mediators—not only enables early and differential diagnosis of neurodegenerative diseases but also directly informs therapeutic strategy, as these same molecules drive secondary injury through mechanisms like proteinjury (secondary protein damage), neuroinflammation, and oxidative stress. This diagnostic–therapeutic link paves the way for personalized CSF-targeted interventions: for instance, Aβ-dominant profiles may warrant anti-Aβ affinity traps or immunoselective nanopheresis, elevated α-synuclein could be addressed with synuclein-specific nanoparticles, and pronounced neuroinflammation might respond best to intrathecal delivery of TNF-α antagonists. Thus, modern CSF-modulating therapies should be viewed not as generic neuroprotective measures, but as precision approaches tailored to the patient’s individual biomarker signature.

**Figure 3:**
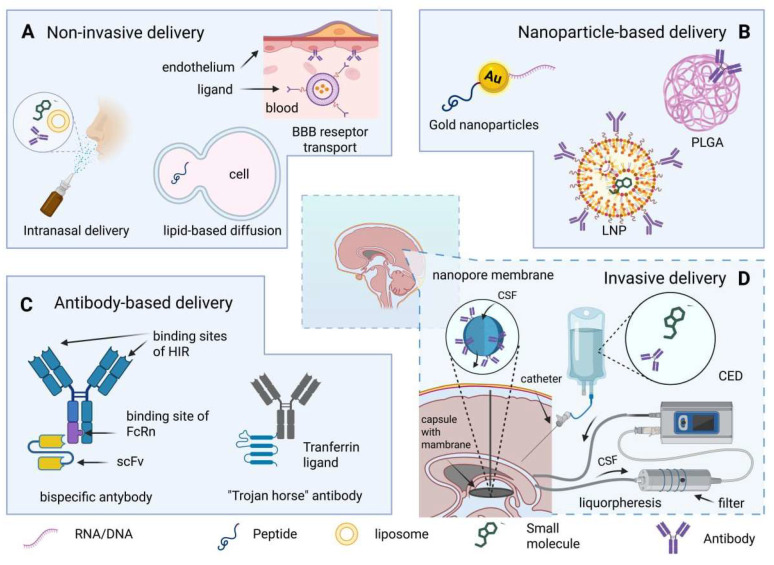
Strategies for therapeutic modulation of CSF composition in neurodegenerative and acute brain disorders. (**A**) Non-invasive delivery exploits endogenous transport systems at the BBB, including receptor-mediated transcytosis (e.g., via transferrin or insulin receptors) and intranasal administration through the olfactory route, enabling selected therapeutics to reach the CSF without surgical intervention. (**B**) Nanoparticle-based delivery utilizes engineered carriers—such as gold nanoparticles, lipid-based nanoparticles, and polymer-based systems—to encapsulate drugs, enhance BBB penetration, provide sustained release, and directly neutralize cytotoxic protein aggregates or inflammatory mediators in the CSF. (**C**) Antibody-based delivery employs bispecific monoclonal antibodies that combine a BBB-targeting domain with a therapeutic domain, enabling targeted delivery of biologics into the CSF and brain parenchyma. (**D**) Invasive delivery encompasses direct CSF access techniques, including intrathecal/ventricular infusion, CED, extracorporeal liquopheresis, and implantable immunoselective nanopheresis devices based on nanoporous membranes that enable “pseudodelivery”—selective removal of pathological factors from the CSF without systemic drug administration. Created with BioRender.com. Created in BioRender. Kachkin, D. (2025) https://BioRender.com/15lv6i5.

Under these conditions, developing effective methods for delivering drugs directly into the CSF or through it into the brain parenchyma becomes not just a technical challenge but a prerequisite for translating pathophysiological knowledge into clinical practice. Modern approaches encompass a wide range of strategies, from molecular “reengineering” of drugs to utilize endogenous transport systems to the use of nanotechnology, bispecific antibodies, and invasive direct-access devices (Figure 3). This section examines key approaches to delivering therapeutic agents to the cerebrospinal space, their mechanisms, limitations, and potential applications in the context of targeted neuroprotection.

### 5.1. Non-Invasive Methods of Delivering Therapeutic Drugs

Effective treatment of neurodegenerative diseases requires the successful delivery of therapeutic agents to the CNS, a process that is severely limited by the presence of the BBB. The BBB, composed of tightly connected endothelial cells lining brain capillaries, restricts passive diffusion from the bloodstream into the brain and prevents the penetration of the vast majority of pharmacological compounds—both low-molecular-weight agents (~98%) and almost all high-molecular-weight biopharmaceuticals [145,146].

Nevertheless, several endogenous transport mechanisms across the BBB can be exploited to design non-invasive therapeutic delivery strategies. The two best-characterized routes are lipid-mediated passive diffusion and carrier- or receptor-mediated active transport (Figure 3).

Lipid-mediated diffusion remains the predominant pathway for a limited subset of small-molecule drugs. To effectively cross the BBB, a compound must satisfy strict physicochemical criteria: a molecular weight below 400 Da and the ability to form no more than 7–8 hydrogen bonds with water molecules—parameters that correlate with high lipophilicity [147]. Only about 6–12% of approved drugs meet these requirements, and most of them are indicated for mood or sleep disorders rather than for neurodegenerative conditions [148]. Another study reported that only 12% of all drugs exhibit activity in the CNS; when agents for affective disorders are excluded, this proportion drops to approximately 1% [149]. CNS-active drugs typically form ≤7 hydrogen bonds with water molecules [150]. Collectively, these findings indicate that the BBB permeability of small-molecule candidates can be reasonably predicted from their chemical structure, particularly molecular weight and hydrogen-bonding capacity.

To overcome these physicochemical constraints, contemporary strategies focus on “re-engineering” therapeutic molecules to exploit endogenous BBB transport systems. Small-molecule drugs may be chemically modified to utilize carrier-mediated transport (CMT) via glucose, amino acid, or organic acid transporters [151,152]. In contrast, large-molecule agents such as proteins, antibodies, or nucleic acids are delivered through receptor-mediated transport (RMT) [153], which employs endogenous receptors including the insulin receptor (HIR) and transferrin receptor (TfR) [154,155,156]. On this basis, “molecular Trojan horses” have been developed—bispecific fusion proteins containing one domain for receptor binding on the BBB and another providing the intended therapeutic function within the brain parenchyma.

An alternative non-invasive route is intranasal delivery, which enables drug penetration into the subarachnoid space via the olfactory pathway (Figure 3). Successful delivery to the CSF through this route requires two sequential events: (1) transport of the compound across the nasal epithelium, and (2) passage across the arachnoid membrane separating the nasal submucosa from the olfactory CSF compartment. These processes are feasible only if the active compound possesses three essential properties: (1) high lipophilicity, (2) low molecular weight, and (3) appropriate ionization [157]. Lipid-soluble small molecules administered intranasally can partially distribute into the olfactory CSF compartment, whereas large or water-soluble compounds generally fail to penetrate in the absence of epithelial disruption.

Experimental evidence suggests that intranasal administration under anesthesia or before sleep can increase the proportion of drug directly reaching the brain, thereby enhancing the rate of its distribution within neural tissue, for example, in the case of caffeine [158].

In summary, despite substantial physiological and structural barriers, understanding the molecular mechanisms governing BBB transport enables the rational design of drugs capable of achieving therapeutic concentrations in the CNS through non-invasive means.

### 5.2. Nanoparticles as a Means of Drug Delivery to CSF

The advent of nanotechnology has opened new prospects for improving drug delivery across the BBB. Among the various nanocarrier systems under investigation, nanoparticles (NPs) have attracted particular attention due to their ability to enhance the transport of therapeutic molecules into the CNS (Figure 3). When considering their use for brain-targeted delivery, the principal challenge lies in ensuring that NPs can penetrate the brain parenchyma and CSF compartments effectively and safely.

NPs offer several advantages over conventional delivery systems, including improved stability of the encapsulated drug, protection from enzymatic degradation, and controlled or sustained release of active compounds [159]. In addition, surface modification allows for targeting specific receptors or transport mechanisms, potentially increasing delivery efficiency while minimizing systemic toxicity.

Currently, several targeted nanoparticle-based approaches are being actively investigated, including systems based on gold NPs, liposomes, polymeric NPs, and lipid nanocarriers. Among these, gold NPs are considered one of the most promising due to their physicochemical properties and biological compatibility.

#### 5.2.1. Gold Nanoparticles

Gold nanoparticles (AuNPs) are typically less than 100 nm in diameter and possess several favorable properties for biomedical applications, including ease of large-scale synthesis, resistance to surface degradation, and high biocompatibility [160]. The uptake of AuNPs by endothelial cells involves multiple mechanisms, including carrier-mediated transport and passive diffusion, as well as endocytic processes, such as receptor-mediated or adsorptive endocytosis, depending on particle size, surface charge, and functionalization [161,162]. Importantly, AuNPs can cross the BBB without compromising its structural integrity [163].

AuNPs have been extensively studied for both therapeutic and diagnostic applications in neurodegenerative diseases. In AD, AuNPs functionalized with amyloid-specific peptides have been shown to inhibit Aβ aggregation and promote targeted delivery of therapeutic agents. In models of AD, functionalized AuNPs demonstrated improved BBB penetration in vitro. In particular, glutathione-encapsulated AuNPs have been developed to prevent Aβ aggregation in AD, suggesting their potential as disease-modifying agents. According to T. O. Lilius et al., combined intracisternal administration of AuNPs with a hypertonic solution enhances the uniformity of AuNP distribution and uptake within the brain parenchyma [164].

Beyond therapeutic applications, AuNPs also hold promise as diagnostic tools. Their optical and surface plasmon properties enable their incorporation into biosensing platforms for detecting pathological proteins. Indeed, recent studies have reported the detection of AuNPs in the CSF of patients with AD and PD, highlighting their potential role as components of complex diagnostic systems [165].

#### 5.2.2. Lipid-Based Nanoparticles

Lipid nanoparticles (LNPs) represent one of the most promising platforms for delivering therapeutic agents to the CSF and CNS. Owing to their biocompatibility, biodegradability, and structural similarity to the lipid constituents of the BBB, LNPs can efficiently traverse or interact with these barrier structures and/or distribute into the CSF following direct administration.

In the context of neurodegenerative disorders, LNPs serve not only as passive carriers but also as modulators of CSF composition. They can reduce the levels of cytotoxic protein aggregates, proinflammatory mediators, and oxidative stress products [166,167]. The main structural components of LNPs—phospholipids, fatty acids, mono- and triglycerides, fatty alcohols, and waxes—closely resemble the lipid matrix of the BBB, facilitating transcellular penetration into the brain [168].

LNPs are generally categorized into three main types: liposomes, solid lipid nanoparticles (SLNs), and nanostructured lipid carriers (NLCs) [169,170,171]. These differ in lipid organization, morphology, loading capacity, particle size, and surface charge characteristics. Importantly, LNPs demonstrate high versatility by encapsulating both hydrophilic and hydrophobic drugs [172].

Despite these advantages, several limitations still impede their large-scale production and clinical translation. Challenges include low long-term stability, complex manufacturing processes, and premature drug release during storage [173,174,175]. Nevertheless, SLNs and NLCs are rapidly emerging as viable diagnostic and therapeutic tools for AD and other CNS pathologies.

Experimental data support their neuroprotective potential. Almuhayawi developed LNPs enriched with pomegranate extract and evaluated them in a rat model of aluminum chloride–induced AD. Pomegranate extract, rich in antioxidants, alkaloids, and tannins, significantly improved cognitive performance, enhanced antioxidant biomarkers in brain homogenates, and reduced Aβ deposition compared with untreated controls [176]. Similarly, Giacomeli demonstrated that curcumin-loaded LNPs enhanced curcumin solubility and bioavailability, resulting in reduced neuroinflammatory markers in hippocampal and cortical tissues, along with improved spatial memory in a mouse model of AD [177].

A recent study by Dara explored the therapeutic application of erythropoietin (EPO) in AD [178]. EPO exerts neuroprotective effects in ischemia, epilepsy, and spinal cord injury and promotes neurogenesis and neuronal survival in PD and AD [179,180,181]. However, due to its hydrophilicity, rapid systemic clearance, and high molecular weight, EPO poorly penetrates the BBB. Encapsulation of EPO in SLNs markedly enhanced its brain delivery in a rat AD model, leading to reduced oxidative stress, diminished Aβ accumulation in the hippocampus, and improved cognitive performance compared with untreated animals [178].

Additionally, LNP-based systems have been investigated for the delivery of small interfering RNA (siRNA), showing promise for both neurodegenerative and oncological CNS diseases. When administered into the CSF, siRNA-loaded LNPs effectively suppress the expression of key pathogenic mediators [182].

An important pharmacokinetic consideration for LNP-based drug delivery is the formation of a protein corona—a layer of adsorbed proteins derived from the biological fluid into which the LNPs are introduced. These fluid-specific protein coronas can substantially alter nanoparticle behavior, including biodistribution, clearance, and target affinity. Therefore, the administration route and local biological milieu must be carefully considered during LNP design and development [183].

#### 5.2.3. Polymer-Based Nanoparticles

Polymer-based nanoparticles are increasingly used in drug delivery systems due to their high biocompatibility, biodegradability, and capacity for controlled and targeted drug release [184,185]. Both synthetic polymers, such as polylactic acid (PLA), polyglycolic acid (PGA), their copolymer PLGA, and polyacrylates, and natural polymers, including chitosan, alginate, and albumin, are commonly employed in their formulation. By fine-tuning particle size, surface charge, and chemical functionalization, polymeric NPs can be engineered to cross the BBB or to be directly administered into the CSF, enabling their distribution throughout the subarachnoid and ventricular spaces.

In neurodegenerative diseases, the relevance of such systems lies not only in their ability to deliver therapeutic molecules, but also in their potential to modulate the pathological composition of the CSF itself.

Among the most studied platforms are biodegradable PLGA-based NPs, which show both BBB permeability and neuroprotective potential [186,187]. Curcumin—a polyphenolic compound with antioxidant and anti-amyloid properties—has demonstrated therapeutic promise in AD, though its application is limited by poor solubility and bioavailability. Mathew developed curcumin-loaded PLGA NPs conjugated with the Tet-1 peptide, facilitating neuronal targeting and BBB translocation [188]. These NPs preserved curcumin’s antioxidant and anti-amyloid properties without cytotoxic effects. Similarly, curcumin-enriched PLGA NPs injected into the hippocampus and subventricular zone of adult rats enhanced neurogenesis by stimulating Wnt/β-catenin pathway activation, leading to improved learning and memory [189,190]. These findings suggest that curcumin–PLGA NPs may promote intrinsic brain repair mechanisms and represent a promising therapeutic approach for AD.

Another compound, andrographolide (AG), a diterpene with potent anti-inflammatory effects, suffers from low bioavailability, limiting its clinical use. To overcome this, Graverini developed AG-loaded NPs based on polyethylene cyanoacrylate (PECA) and human serum albumin (HSA) [191]. Using a human brain microvascular endothelial cell model (hCMEC/D3), Guccione demonstrated that HSA-based NPs significantly enhanced AG permeability across the BBB while preserving barrier integrity, whereas PECA NPs caused cellular disruption [192]. These data support albumin-based nanocarriers as safer and more efficient vehicles for AG delivery to the brain.

Quercetin (QT), another bioactive polyphenol, has also been successfully encapsulated in PLGA NPs. PLGA–QT NPs inhibited and disaggregated Aβ42 fibrils, improved neuronal survival, and mitigated Zn^2+^/Aβ42-induced neurotoxicity in vitro. In APP/PS1 transgenic mice, these NPs improved memory and cognitive performance [193]. Similarly, phytol-encapsulated PLGA NPs demonstrated a spherical nanoscale morphology, sustained drug release, and robust acetylcholinesterase inhibition. In neuronal cell cultures, these NPs disrupted Aβ aggregates and exhibited moderate cytotoxicity, further supporting their potential in AD therapy [194]. Collectively, these studies indicate that phytochemical-loaded polymeric nanocarriers can cross the BBB and modulate multiple pathogenic processes characteristic of neurodegeneration.

Importantly, when administered intrathecally or intraventricularly, polymeric NPs exhibit prolonged circulation within the CSF, allowing for sustained release of therapeutic agents while minimizing systemic exposure. This pharmacokinetic feature is particularly valuable for chronic neurodegenerative disorders, which require long-term modulation of CSF composition, especially the reduction in cytotoxic protein aggregates, proinflammatory cytokines, and oxidative stress products.

Thus, polymer-based NPs function not merely as inert delivery vehicles, but as active therapeutic platforms capable of directly modifying the pathological milieu of the CSF and thereby interrupting the cascade of secondary injury in neurodegenerative diseases.

#### 5.2.4. Dendrimers

Dendrimers—highly branched, monodisperse synthetic macromolecules with a well-defined three-dimensional architecture—represent a promising platform for targeted drug delivery to the cerebrospinal space [195]. Owing to their precisely controlled structure, internal cavities, and abundant surface functional groups, dendrimers can be engineered both to encapsulate active substances and to facilitate their transport across the BBB or efficient distribution within the CSF [196]. Their relevance is particularly evident in the context of neurodegenerative diseases, where neutralization of cytotoxic protein aggregates circulating in the CSF is a major therapeutic objective.

Polyamidoamine (PAMAM)-based dendrimers have been shown to exert a pronounced anti-amyloidogenic effect: they not only prevent the formation of Aβ fibrils but also promote the disaggregation of preformed oligomers [112,197,198,199]. This activity is of particular importance, as soluble forms of Aβ in the CSF are key mediators of extracellular proteotoxic damage to neurons. Similar effects have been demonstrated for α-synuclein, a major pathogenic factor in PD and related synucleinopathies: cationic carbosilane dendrimers effectively inhibit α-synuclein fibrillation and protect neuronal cells from rotenone-induced toxicity [200].

A notable advantage of dendrimers lies in their capacity for selective accumulation within regions of CNS pathology, including sites of inflammation and degeneration. This targeted distribution enhances therapeutic efficacy while minimizing systemic toxicity. To improve biocompatibility and prolong systemic circulation, dendrimer surfaces are often chemically modified, most commonly through PEGylation, acetylation, or glycosylation [201,202]. Among these, structures terminating in carboxyl or hydroxyl groups are considered the safest, as they minimize nonspecific membrane interactions. For example, dendritic polyglycerol (dPG) microspheres loaded with dimethyl fumarate and curcumin demonstrated favorable sustained release and low cytotoxicity in vitro, suggesting potential applicability in multiple sclerosis therapy [203].

Although the majority of current studies remain limited to in vitro and preclinical animal models, the available data underscore the potential of dendrimers to act not only as delivery vehicles but also as active therapeutic agents capable of directly modulating the pathological composition of the CSF. Further research on dendrimer biocompatibility, pharmacokinetics, and targeting efficiency could facilitate the development of novel strategies for “cleansing” the CSF of cytotoxic protein aggregates and restoring its homeostasis in chronic neurodegenerative disorders.

### 5.3. Monoclonal Therapeutic Antibodies

Monoclonal antibodies represent a powerful class of therapeutic agents capable of specifically neutralizing key mediators of secondary injury circulating in the CSF or localized within brain tissue (Figure 3). Their clinical potential in neurodegenerative diseases has historically been limited by poor permeability across the BBB; less than 0.1% of the systemically administered dose typically reaches the CSF. This limitation necessitates the use of high doses, which increases the risk of adverse effects, including amyloid-related imaging abnormalities (ARIA), such as cerebral edema and microhemorrhages [204].

To overcome this barrier, recombinant bifunctional IgG fusion proteins have been developed. These molecules consist of two functional domains: a transport domain, which serves as a molecular “Trojan horse” targeting an endogenous BBB receptor to induce receptor-mediated transcytosis into the brain, and a therapeutic domain, which exerts the pharmacological effect once inside the CNS. Common transport targets include the human insulin receptor (HIR), transferrin receptor (TfR), and the CD68 glycoprotein, part of the LAT1 amino acid transporter. The therapeutic domain is designed to act against a specific pathological target, with indications corresponding to primary CNS disorders [205].

Experimental studies have demonstrated the efficacy of such constructs in models of both acute and chronic CNS pathology. For example, a bispecific antibody targeting oligomeric α-synuclein (RmAb38E2) fused with two single-chain variable fragment (scFv) fragments against TfR (8D3) effectively crossed the BBB in a mouse model of synucleinopathy. Treatment reduced α-synuclein oligomer accumulation in the brain, promoted microglial activation, and showed improved CNS penetration compared to conventional antibodies lacking a transport domain [206].

Similar strategies have been applied to lysosomal storage disorders such as Hurler syndrome (MPS I) and Hunter syndrome (MPS II), where chimeric monoclonal antibodies fused with the deficient enzymes (IDUA or IDS) successfully penetrated the BBB in non-human primates and reduced substrate accumulation in patient-derived fibroblasts. These fusion proteins represent the first biotherapeutics targeting the BBB to undergo full clinical trials in humans [205,207].

#### 5.3.1. Bispecific Therapeutic Antibodies

In addition to their transport and therapeutic functions, chimeric antibodies can be modified for additional functions. For example, anti-Aβ mAb for AD immunotherapy was modified to penetrate the BBB in both directions [208]. This process involves three steps: transport of the anti-Aβ antibody from the blood to the brain across the BBB; binding and disaggregation of Aβ fibrils in the brain; and efflux of the anti-Aβ antibody from the brain back into the blood. This trifunctional molecule, HIRMAb-Aβ-scFv, includes a transport domain, i.e., HIRMAb; a therapeutic domain, i.e., a single-chain anti-Aβ antibody monomer (scFv) fused to the carboxyl terminus of the HIRMAb heavy chain; and a neonatal Fc receptor or FcRn binding site located in the CH2 and CH3 domains of human IgG, which mediates efflux of HIRMAb-Aβ-scFv from the brain. The chimeric bifunctional antibody HIRMAb-Aβ-scFv was constructed by introducing scFv targeting the Aβ1-28 peptide at the C-terminus of the HIRMAb heavy chain via a short Ser-Ser linker. The tetravalent bifunctional mAb retained high affinity for both Aβ and the BBB insulin receptor. The pharmacokinetics and biodistribution of the fused HIRMAb-Aβ-scFv mAb were studied in rhesus monkeys using a [125I]-labeled test preparation and compared with a control preparation that included a 3H-labeled original murine mAb targeting Aβ (Mab-Aβ) [208]. Following administration, no measurable decrease in the blood concentration of the control mAb-Aβ was observed. In contrast, rapid clearance of the 125I-HIRMAb-Aβ-scFv fusion antibody from the blood was observed, as this fusion protein targets the brain and peripheral organs expressing the insulin receptor. Thus, there was a global distribution of the fusion mAb in the brain, with preferential uptake in gray matter compared to white matter. Capillary depletion assay revealed a high volume of distribution in the brain of the bifunctional fusion mAb, demonstrating that HIRMAb-Aβ-scFv crossed the BBB into the postcapillary compartment of the brain. On the other hand, the control mouse mAb-Aβ had a distribution volume in the brain of 10 μL/g brain, which is approximately the same as the cerebral arterial blood volume, confirming that the control mAb-Aβ does not penetrate the BBB, remaining in the circulatory compartment of primates.

#### 5.3.2. Decoy-IgG Receptor Fusion Proteins

Other potential new drugs for the treatment of brain diseases include decoy receptors [208]. A decoy receptor is formed by the extracellular domain (ECD) of a receptor fused to the amino terminus of the Fc region of human IgG1. A good example is the ECD of the tumor necrosis factor receptor (TNFR) type II fused to an antibody to the insulin receptor—HIRMAb-TNFR [209]. TNFα, being a proinflammatory cytokine, is a cause of neuroinflammation, and its high levels have been recorded in neurodegenerative conditions such as stroke [210], traumatic injuries of the brain and spinal cord [211,212], neurodegeneration [213] and depression [214]. Blocking TNFα with a specific antagonist resulted in reduced neuronal death, neuroinflammation, and cognitive dysfunction [215]. A chimeric decoy protein was constructed by inserting cDNA encoding human TNFR ECD into the C-terminus of the HIRMAb heavy chain via a serine bridge [216]. Brain uptake of HIRMAb-TNFR was studied in rhesus macaques and compared with that of TNFR:Fc. In the 6-hydroxydopamine model of PD, mouse TfRMAb-TNFR exhibited neuroprotective effects. In a mouse stroke model with reversible middle cerebral artery occlusion, TfRMAb-TNFR also exerted neuroprotective effects, significantly reducing stroke volumes in the cerebral hemispheres, cortex, and subcortical areas. We have compiled information on the use of brain-penetrating IgG fusion proteins in Table 2.

Based on the above data, virtually any protein-based therapeutic can be converted into a brain-penetrating IgG fusion protein. These IgG fusion proteins comprise a transport domain that targets endogenous BBB transporters, inducing receptor-mediated transport into the brain and CSF, and a therapeutic domain that exerts its pharmacological action in the brain after transport across the BBB.

### 5.4. Invasive Technologies for Drug Delivery into the CSF

In neurodegenerative diseases, TBI, and stroke, pathogenesis is driven not only by the accumulation of cytotoxic agents within the brain parenchyma but also by their prolonged presence in the CSF, where they mediate “proteinjury”—extracellular proteotoxic damage. Under these conditions, noninvasive delivery methods are often insufficient to achieve therapeutically relevant drug concentrations in the CSF. Invasive techniques, by contrast, provide direct access to the CSF, enabling both drug delivery and active modulation of its pathological composition (Figure 3).

The most widely applied clinical approaches involve intrathecal and intraventricular infusions using implantable devices, such as Ommaya reservoirs or programmable intrathecal pumps. These systems ensure a stable supply of therapeutic agents directly into the CSF, which is particularly critical for chronic neurodegenerative disorders that require long-term modulation of CSF composition [224,225].

Despite their efficacy, these methods carry risks, including infection, granuloma formation, disruption of CSF dynamics, and ependymal damage when high local drug concentrations are reached [226].

Recently, innovative invasive strategies have focused not solely on delivering exogenous substances but on actively “cleansing” the CSF of cytotoxic factors. These approaches are consistent with the central concept of this review: the CSF is not merely a passive conduit for pathological processes but a dynamic environment that can be therapeutically modulated to interrupt the cascade of secondary injury.

#### 5.4.1. Immunoselective Nanopheresis and “Pseudodelivery” Through Nanoporous Membranes

Central to emerging invasive strategies are nanoporous membranes—artificial barriers with precisely controlled pore sizes (1–10 nm) that selectively allow the passage of molecules according to their mass, three-dimensional conformation, and charge [227,228]. These membranes form the basis of implantable devices designed to selectively remove pathological components from the CSF without the need for systemic administration of therapeutic agents [229,230,231,232].

The application of nanoporous membranes in immunoselective nanopheresis (a process for the selective removal of molecules from CSF using nanoporous membranes and immobilized affinity traps) systems is particularly significant. In these devices, the membrane encloses a capsule containing monoclonal antibodies or other affinity traps directed against specific toxic targets, such as Aβ oligomers or α-synuclein. The membrane permits the entry of low- and medium-molecular-weight toxic agents from the CSF, where they are bound and neutralized, while retaining therapeutic proteins within the capsule to prevent systemic dissemination [233].

Experimental studies in APP/PS1 transgenic mice have demonstrated that implantation of devices loaded with antibodies against Aβ significantly reduces soluble Aβ levels in the CSF and diminishes cortical plaque burden [234,235]. This approach, termed “pseudodelivery,” minimizes systemic side effects, reduces the required drug dose, and provides long-term modulation of CSF composition [236].

Furthermore, nanoporous membranes can be functionalized to selectively remove specific pathological molecules from proinflammatory cytokines to tau aggregates opening avenues for personalized therapy guided by the molecular profile of a patient’s CSF.

#### 5.4.2. Extracorporeal Liquopheresis as a Method of Systemic CSF Modulation

Another promising strategy is extracorporeal liquopheresis, in which CSF is withdrawn from the subarachnoid or ventricular space, passed through an external filtration circuit, and returned. Unlike plasmapheresis, liquopheresis allows for direct reduction of pathological proteins, cytokines, and autoantibodies within the CSF compartment [237].

Although most studies have focused on autoimmune disorders, such as Guillain–Barré syndrome, data suggest that this approach may also be applicable to neurodegenerative diseases. For instance, in vitro filtration of CSF from Alzheimer’s disease patients reduces its neurotoxicity in neuronal cultures [238]. Similarly, CSF from Guillain–Barré patients subjected to liquopheresis loses its ability to induce peripheral nerve demyelination in animal models [239].

Liquopheresis is particularly relevant within the framework of proteinjury: the removal of toxic aggregates from the CSF interrupts their diffuse spread and protects distant neuronal populations from secondary damage. The method can be adapted for non-selective filtration (e.g., ultrafiltration membranes) or for targeted removal of specific molecules using affinity sorbents.

#### 5.4.3. Convection-Enhanced Delivery

Convection-enhanced delivery (CED) is an invasive technique in which a therapeutic agent is infused directly into the brain parenchyma or near the ventricular system under controlled pressure, generating a convective flow that facilitates drug distribution over substantial distances from the infusion site. While initially developed for local brain tissue delivery, CED has gained attention for its potential effects on CSF composition in chronic neurodegenerative processes.

Unlike passive diffusion, which rapidly loses efficacy with distance, CED enables the delivery of therapeutic agents, including neurotrophins, antibodies, and NPs to large regions of the brain, including areas adjacent to the subarachnoid and ventricular spaces [240]. This is particularly important in the context of proteinjury, where cytotoxic aggregates (Aβ, α-synuclein, tau) circulate in the CSF and cause diffuse secondary damage. Administration of affinity traps or disaggregating agents via CED can neutralize pathological proteins in the parenchyma and create a concentration gradient that promotes their efflux into the CSF for subsequent clearance [241].

However, in practice, CED efficacy is limited. Primate studies indicate that even with volumetric infusion, drug concentrations (e.g., GDNF) decrease logarithmically within millimeters of the catheter tip, suggesting that diffusion dominates over convection in dense neuronal tissue. High local concentrations near the catheter can also induce ependymal toxicity and disrupt CSF flow [242].

Of particular interest is CED’s effect on the intraventricular system. In a study by Bander et al., patients with diffuse pontine glioma who received CED radioimmunotherapy exhibited a persistent increase in lateral ventricular volume lasting 30 days post-procedure, indicating that parenchymal infusion near the ventricles can mechanically and osmotically influence CSF circulation [243]. Depending on parameter settings, such effects may either disrupt or facilitate the “flushing” of CSF from pathological agents.

To enhance CED efficacy, combination strategies with nanotechnology are being explored. For example, nanofiber precursors (NFPs) can serve as carriers to prolong drug residence in brain tissue and reduce systemic exposure. Such hybrid systems could provide local neuroprotection and act as a “therapeutic reservoir” releasing agents capable of modulating CSF composition [244].

Thus, despite technical and biological limitations, CED remains a key invasive tool, enabling direct intervention in the CSF. Its therapeutic potential lies not in global drug delivery, but in the creation of localized gradients that facilitate neutralization and removal of cytotoxic factors circulating in the CSF in neurodegenerative diseases [245].

Despite compelling pathophysiological evidence for the role of CSF in exacerbating secondary injury, direct evidence for the therapeutic efficacy of strategies aimed at modulating its composition remains limited. Experimental models have demonstrated reduced levels of pathological aggregates (e.g., Aβ or α-synuclein) and improved behavioral performance using approaches such as immunoselective nanopheresis, intraventricular decoys for toxic proteins, or extracorporeal liquophoresis. However, convincing data on histological or functional recovery from clinical trials are still lacking; most studies are in the preclinical stage or include small cohorts without blinded assessment. Therefore, future studies should be designed to minimize the risk of bias: with prespecified endpoints including quantitative assessment of CSF toxicity, electrophysiological parameters of neuronal and synaptic function, and standardized behavioral tests with independent assessment. Only such a strategy will allow us to match a compelling causal framework with measurable clinical benefit.

Thus, invasive technologies offer a fundamentally new approach to treating neurodegenerative diseases: rather than focusing solely on “delivering a drug to the brain” they enable direct intervention in the CSF as a pathological milieu. By modulating its molecular composition, these methods can interrupt the cascade of secondary damage at a systemic level. Comparative characteristics of such methods are presented below in Table 3.

## 6. Conclusions

Current data convincingly demonstrate that CSF is far from a passive environment, but a dynamic and functionally active system that plays a central role in the pathogenesis of neurodegenerative diseases, TBI, and stroke. It is through the CSF that key factors of secondary damage are transmitted: oxidative stress, excitotoxicity, neuroinflammation, proteotoxic aggregates, and exosomes, which carry pathological signals between brain regions. Furthermore, barrier structures (the blood–brain and blood–cerebrospinal fluid barriers, the choroid plexus, and the ependyma) not only regulate the composition of the CSF but also become targets for damaging agents, creating a vicious cycle that accelerates neurodegeneration.

Despite promising results from preclinical studies, most current therapeutic strategies aimed at modulating CSF composition face fundamental limitations. Some approaches, such as systemic monoclonal antibodies against Aβ or α-synuclein, demonstrate modest efficacy with a significant risk of amyloid-associated complications and require high doses due to extremely low permeability across the BBB. Nanoparticles and intranasal systems, although bypassing some barriers, often suffer from unpredictable pharmacokinetics, nonspecific uptake by the reticuloendothelial system, and potential neurotoxicity with chronic use. Meanwhile, invasive methods—from intraventricular infusions to liquopheresis—remain poorly understood in terms of their long-term impact on CSF homeostasis and carry risks associated with disruption of CSF circulation or loss of endogenous neurotrophic factors. In this regard, a promising direction emerging from this review is the transition from “rough” neutralization of pathological agents to targeted, personalized correction of CSF “toxicity”—using immunoselective nanoporous implants or hybrid delivery systems combining nanocarriers with bispecific “Trojan horses.” Such approaches, focused not on global suppression of pathology but on restoring CSF molecular balance, can provide sustained neuroprotection with minimal systemic side effects, especially when combined with biomarker monitoring. This dual nature of CSF as a source of pathology and as a diagnostic window makes it a unique therapeutic target. On the one hand, the profile of proteins, cytokines, neuronal, and glial markers in CSF enables highly accurate diagnosis and differentiation of neurodegenerative conditions in the early stages. On the other hand, modulating CSF composition, whether through neutralization of toxic aggregates, suppression of inflammation, or enhancement of clearance systems, opens up prospects for neuroprotection even months and years after the initial injury.

However, realizing these potentials directly depends on effective strategies for delivering therapeutic agents into the cerebrospinal space. Modern approaches—from bispecific antibodies and intelligent nanocarriers to invasive technologies such as immunoselective liquopheresis and nanoporous “pseudodelivery” implants—demonstrate a shift from systemic therapy to personalized, locally targeted interventions. This synthesis of pathophysiological understanding, diagnostic accuracy, and innovative delivery methods forms the basis for a new paradigm in the treatment of neurodegenerative disorders: therapy that targets not only symptoms but also the brain’s molecular environment through CSF regulation.

## Figures and Tables

**Table 1:** The table presents information on CSF biomarkers that can be used to diagnose various neuropathologies.

Disease	Marker	Characteristics of Changes in CSF Content (Compared to Healthy Patients) and References
Alzheimer’s disease	Aβ42	Reduction in content, one of the main markers
	Tau (total and phosphorylated)	Increased content, one of the main markers [125,126]
	NfL и NfH; GFAP; BACE1; APP; VSNL1	Increased content, are candidates for the role of additional markers [127,128,129,130]
Parkinson’s disease	α-synuclein	Decreased levels, a leading candidate for biomarker [131,132]
	NfL	There are no changes, but it is reduced in comparison with atypical parkinsonian syndromes [132]
	Aβ42	Reduction in content [132]
	Tau	Increase in content [133]
Atypical parkinsonian syndromes	NfL and NfH	The nature of the changes depends on the specific disease and are considered the most promising markers [131,132]
	Aβ40 and Aβ42, tau, α-synuclein, tau/α-synuclein ratio	The nature of the changes depends on the specific disease [134]
Amyotrophic lateral sclerosis	NfL and NfH	Increase in content [135]
	Complement 3	Increase in content [136]
	SOD1, metalloproteinases 2 and 9 (MMP 2 and 9)	Data on the nature of the changes is contradictory [137]
	TDP-43	Increase in content [138]
	A combination of transthyretin, cystatin C, and the carboxyl-terminal fragment of neuroendocrine protein 7B2	Increase in content [139]
Huntington’s disease	Huntingtin (mutant form)	Appearance in CSF (absent in healthy people) [120]
	Ubiquitin	Increase in content [140]
	Tau, NfL	Increase in content [141]
Creutzfeldt-Jakob disease	Protein 14-3-3	Increased content, the main biomarker of CSF [142]
	Tau, ratio of total and phosphorylated tau	An increase in the content is considered as an additional marker of the disease [143]
	PrPSc	Appearance in CSF (absent in healthy people) [144]

**Table 2:** Human brain-penetrating IgG fusion proteins.

Fused Protein IgG ^1^	Therapeutic Domain	Disease of the Nervous System	Therapeutic Effect	References
RmAb38E2-scFv8D3	Antibody to the oligomeric form of α-synuclein	PD (synucleinopathy)	Reduction of α-synuclein oligomer accumulation in a mouse model of synucleinopathy	[206]
HIRMAb-IDUA (valanafusp alpha)	Iduronidase (IDUA)	Hurler syndrome (MPS I)	Decreased glucosaminoglycan production in fibroblasts from patients with MSP I	[216]
HIRMAb-IDS	Iduronate-2-sulfatase (IDS)	Hunter syndrome (MPS II)	Reduction of polysaccharide accumulation	[217]
BBB-mGluR1	Antagonist of metabotropic glutamate receptor type 1	Chronic pain of inflammatory origin	Suppression of thermal hyperalgesia in a rat model of chronic pain	[218]
Bispecific antibody HIRMAb-Aβ	Anti-Aβ single-chain Fv antibody (scFv)	AD *	Reduction of Aβ deposits in a transgenic mouse model	[208]
HIRMAb-TNFR	Tumor necrosis factor decoy receptor (TNFR)	PD, ALS, AD, stroke *	Effective penetration of the BBB and clearance from the brain in rhesus macaquesReduction of TNFα-mediated cell death in an in vitro model of actinomycin D action	[219]
HIRMAb-EPO	Erythropoietin (EPO)	PD, AD, Friedreich’s ataxia *	Reduction of ischemic lesion volume in the rat middle cerebral artery occlusion model	[220]
HIRMAb-GDNF	Glial cell-derived neurotrophic factor (GDNF)	PD, stroke, drug/EtOH addiction *	Reduction of ischemic lesion volume in the rat middle cerebral artery occlusion model	[221]
HIRMAb-BDNF	Brain-derived neurotrophic factor (BDNF)	Stroke, recovery (regenerative processes) of the nervous system *	Crossing the BBB was confirmed in isolated capillaries.Reduction of cell death in an in vitro hypoxia model.	[222]
CD98hc-TrkB	Neurotrophin receptor antibodies (TrkB)	AD, PD, Huntington’s disease	Blockade of TrkB-mediated signaling in vivo	[223]

^1^ The transport domain of these human fusion proteins is a monoclonal antibody directed against the human BBB insulin receptor (HIRMAb) or transferrin receptor (TfRMAb), or the CD68 glycoprotein, which is part of the LAT1 amino acid transporter. The therapeutic target of the fusion protein and its use are described for the corresponding IgG fusion protein. * Indication associated with primary CNS disease.

**Table 3:** Neurotherapeutic drug delivery strategies: efficacy, risks, and development stages.

Delivery Method	Mechanism	Route of Administration	Estimated Concentration/Efficiency	Risks	Clinical Status
Nanoparticles	Lipophilic diffusion, adsorptive-mediated transcytosis of NPs with therapeutic cargo	Intravenous, intranasal, oral	0.001% to 0.01% of the injected dose	Hepatotoxicity, chronic neuroinflammation, BBB disruption, complement activation-related pseudoallergy, burst drug release and local neurotoxicity	Pre-clinical
Engineered antibodies	Target inactivation with specific antibodies passing through BBB with the help of molecular “Trojan horse” targeting an endogenous BBB receptor	Intravenous, intranasal	0.5% to 3% of plasma concentration, 5–10 times more compared to passive immunization	Immunogenicity, unfavorable pharmacokinetics, cytokine release syndrome, anemia	Phase I/II
Nanopheresis	Target inactivation through continuous CSF filtration through nanopore membrane filled with drug e.g., antibody	Invasive through intrathecal capsule	Reduction of 30–50% of therapeutic target	Intracranial hemorrhage, inflammatory response, loss of essential biomolecules	Pre-clinical
Liquorpheresis	CSF filtering—protein depletion total protein	Invasive: intrathecal CSF Filtration	20–40% of protein decrease in CSF	Loss of essential biomolecules, protein aggregation, hydrodinamic stress, intracranial hypotension,	Phase I
Convection-enchased delivery	Direct delivery into parenchyma with a drain	Invasive through cerebral catheter	High concentration of drug near the catheter but decreases logarithmically within millimeters of the catheter tip	Backflow (reflux), local neurotoxicity	Phase I/II

## Data Availability

No new data were created or analyzed in this study. Data sharing is not applicable to this article.

## Abbreviations

The following abbreviations are used in this manuscript:

AD	Alzheimer’s disease
ALS	Amyotrophic lateral sclerosis
AMPA	α-Amino-3-hydroxy-5-methyl-4-isoxazolepropionic acid
APP	Amyloid-beta precursor protein
ARIA	Amyloid-related imaging abnormalities
AuNPs	Gold nanoparticles
BACE1	Beta-site amyloid precursor protein-cleaving enzyme 1
BBB	Blood-brain barrier
BDNF	Brain-derived neurotrophic factor
CNS	Central nervous system
CSF	Cerebrospinal fluid
DJ-1	Protein deglycase DJ-1 (Parkinson’s disease protein 7)
ECD	Extracellular domain
EPO	Erythropoietin
FcRn	Neonatal Fc receptor
GAPDH	Glyceraldehyde-3-phosphate dehydrogenase
GFAP	Glial fibrillary acidic protein
GDNF	Glial cell line-derived neurotrophic factor
HIR	Human insulin receptor
HIRMAb	Human insulin receptor monoclonal antibody
IDUA	α-L-iduronidase
IDS	Iduronate-2-sulfatase
IgG	Immunoglobulin G
IL	Interleukin
LFP	Lateral fluid percussion (used contextually in TBI models)
LNPs	Lipid-based nanoparticles
mPTP	Mitochondrial permeability transition pore
MPS	Mucopolysaccharidosis
NDDs	Neurodegenerative diseases
NfH	Neurofilament heavy chain
NfL	Neurofilament light chain
NMDA	N-Methyl-D-aspartate
NPs	Nanoparticles
PLGA	Poly(lactic-co-glycolic acid)
PrPSc	Scrapie form of prion protein
RAGE	Receptor for advanced glycation end-products
ROS	Reactive oxygen species
RNS	Reactive nitrogen species
RT-QuIC	Real-time quaking-induced conversion
scFv	Single-chain variable fragment
SLNs	Solid lipid nanoparticles
SOD1	Superoxide dismutase 1
TBI	Traumatic brain injury
TDP-43	TAR DNA-binding protein 43
TfR	Transferrin receptor
TfRMAb	Transferrin receptor monoclonal antibody
TNF-α	Tumor necrosis factor alpha
TNFR	Tumor necrosis factor receptor
VSNL1	Visinin-like protein 1
